# Supplementary material for: MELK is not necessary for the proliferation of basal-like breast cancer cells
Source: eLife. 2017 Sep 19;6:e26693. doi: 10.7554/eLife.26693 (PMC5605198; doi:10.7554/eLife.26693)
Supplement: Figure 1—source data 1. [file elife-26693-fig1-data1.docx]

**Figure 1—Source Data 1. Kinase profiling of OTSSP167 and HTH-01-091 by the International Center for Kinase Profiling (ICKP)**

|  | OTSSP167 (1 μM) | | HTH-01-091 (1 μM) | |  |  | OTSSP167 (1 μM) | | HTH-01-091 (1 μM) | |
| --- | --- | --- | --- | --- | --- | --- | --- | --- | --- | --- |
| Kinase | % AR^a^ | SD^b^ | % AR^a^ | SD^b^ |  | Kinase | % AR^a^ | SD | % AR^a^ | SD^b^ |
| MKK1 | 3 | 0 | 62 | 5 |  | NEK2a | 59 | 11 | 100 | 7 |
| MKK2 | 10 | 1 | 75 | 9 |  | NEK6 | 92 | 3 | 95 | 8 |
| MKK6 | 80 | 2 | 99 | 10 |  | IKK beta | 41 | 1 | 104 | 13 |
| ERK1 | 88 | 1 | 119 | 17 |  | IKK epsilon | 2 | 0 | 92 | 5 |
| ERK2 | 90 | 5 | 93 | 7 |  | TBK1 | 6 | 1 | 88 | 17 |
| ERK5 | 4 | 1 | 70 | 12 |  | PIM1 | 10 | 1 | 1 | 0 |
| JNK1 | 10 | 1 | 97 | 3 |  | PIM2 | 0 | 0 | 2 | 0 |
| JNK2 | 16 | 1 | 100 | 3 |  | PIM3 | 1 | 0 | 1 | 1 |
| JNK3 | 13 | 1 | 101 | 1 |  | SRPK1 | 10 | 1 | 94 | 13 |
| p38 alpha | 10 | 1 | 84 | 2 |  | EF2K | 114 | 5 | 85 | 13 |
| p38 beta | 6 | 0 | 85 | 9 |  | EIF2AK3 | 7 | 0 | 114 | 18 |
| p38 gamma | 12 | 5 | 86 | 2 |  | HIPK1 | 0 | 0 | 24 | 3 |
| p38 delta | 4 | 1 | 89 | 0 |  | HIPK2 | 2 | 2 | 13 | 6 |
| ERK8 | 1 | 1 | 24 | 4 |  | HIPK3 | 0 | 0 | 25 | 4 |
| RSK1 | 3 | 2 | 48 | 4 |  | CLK2 | 0 | 0 | 11 | 0 |
| RSK2 | 1 | 1 | 62 | 11 |  | PAK2 | 74 | 10 | 88 | 24 |
| PDK1 | 4 | 1 | 102 | 0 |  | PAK4 | 9 | 2 | 79 | 1 |
| PKB alpha | 28 | 1 | 101 | 3 |  | PAK5 | 19 | 1 | 73 | 5 |
| PKB beta | 24 | 2 | 88 | 5 |  | PAK6 | 29 | 3 | 89 | 4 |
| SGK1 | 12 | 0 | 72 | 4 |  | MST2 | 7 | 2 | 45 | 0 |
| S6K1 | 2 | 0 | 81 | 2 |  | MST3 | 5 | 0 | 93 | 1 |
| PKA | 12 | 0 | 112 | 24 |  | MST4 | 5 | 1 | 96 | 3 |
| ROCK 2 | 49 | 1 | 82 | 5 |  | GCK | 0 | 0 | 16 | 1 |
| PRK2 | 2 | 0 | 77 | 0 |  | MAP4K3 | 1 | 0 | 55 | 1 |
| PKC alpha | 7 | 1 | 79 | 17 |  | MAP4K5 | 3 | 1 | 32 | 0 |
| PKC gamma | 1 | 0 | 71 | 7 |  | MINK1 | 2 | 1 | 38 | 1 |
| PKC zeta | 19 | 1 | 96 | 9 |  | MEKK1 | 96 | 19 | 111 | 8 |
| PKD1 | 6 | 0 | 79 | 11 |  | MLK1 | 1 | 1 | 35 | 0 |
| STK33 | 10 | 1 | 26 | 1 |  | MLK3 | 2 | 1 | 54 | 0 |
| MSK1 | 5 | 0 | 65 | 2 |  | TESK1 | 51 | 9 | 95 | 0 |
| MNK1 | 28 | 0 | 109 | 1 |  | TAO1 | 3 | 0 | 18 | 1 |
| MNK2 | 13 | 2 | 103 | 2 |  | ASK1 | 7 | 2 | 90 | 1 |
| MAPKAP-K2 | 14 | 4 | 98 | 10 |  | TAK1 | 1 | 0 | 22 | 0 |
| MAPKAP-K3 | 94 | 0 | 89 | 5 |  | IRAK1 | 3 | 1 | 86 | 6 |
| PRAK | 16 | 0 | 78 | 4 |  | IRAK4 | 7 | 1 | 85 | 7 |
| CAMKK beta | 2 | 1 | 91 | 54 |  | RIPK2 | 4 | 0 | 3 | 1 |
| CAMK1 | 15 | 1 | 93 | 5 |  | OSR1 | 45 | 3 | 93 | 4 |
| SmMLCK | 6 | 3 | 8 | 1 |  | TTK | 3 | 2 | 53 | 3 |
| PHK | 3 | 4 | 74 | 4 |  | MPSK1 | 5 | 0 | 107 | 11 |
| DAPK1 | 1 | 0 | 15 | 1 |  | WNK1 | 111 | 5 | 101 | 4 |
| CHK1 | 11 | 1 | 86 | 8 |  | ULK1 | 32 | 0 | 93 | 1 |
| CHK2 | 1 | 0 | 34 | 0 |  | ULK2 | 4 | 0 | 105 | 15 |
| GSK3 beta | 0 | 0 | 86 | 4 |  | TGFBR1 | 16 | 1 | 102 | 10 |
| CDK2-Cyclin A | 2 | 0 | 67 | 16 |  | Src | 5 | 3 | 54 | 2 |
| CDK9-Cyclin T1 | 4 | 1 | 92 | 4 |  | Lck | 0 | 0 | 35 | 5 |
| PLK1 | 3 | 0 | 95 | 0 |  | CSK | 9 | 6 | 76 | 2 |
| Aurora A | 10 | 1 | 98 | 7 |  | YES1 | 18 | 9 | 32 | 0 |
| Aurora B | 14 | 1 | 35 | 2 |  | ABL | 1 | 0 | 70 | 3 |
| TLK1 | 58 | 1 | 95 | 6 |  | BTK | 7 | 3 | 45 | 5 |
| LKB1 | 83 | 8 | 100 | 11 |  | JAK2 | 3 | 1 | 39 | 2 |
| AMPK | 2 | 0 |  |  |  | SYK | 7 | 1 | 112 | 4 |
| AMPK (hum) | 1 | 0 | 34 | 3 |  | ZAP70 | 78 | 13 | 98 | 15 |
| MARK1 | 31 | 1 | 97 | 1 |  | TIE2 | 3 | 0 | 71 | 3 |
| MARK2 | 23 | 1 | 94 | 3 |  | BRK | 8 | 3 | 96 | 4 |
| MARK3 | 13 | 3 | 94 | 2 |  | EPH-A2 | 7 | 0 | 66 | 8 |
| MARK4 | 14 | 0 | 95 | 0 |  | EPH-A4 | 1 | 1 | 63 | 3 |
| BRSK1 | 2 | 0 | 85 | 9 |  | EPH-B1 | 11 | 2 | 122 | 7 |
| BRSK2 | 2 | 2 | 125 | 2 |  | EPH-B2 | 1 | 1 | 70 | 2 |
| MELK | 6 | 1 | 13 | 0 |  | EPH-B3 | 18 | 7 | 99 | 23 |
| NUAK1 | 0 | 2 | 66 | 4 |  | EPH-B4 | 2 | 0 | 104 | 17 |
| SIK2 | 2 | 0 | 79 | 2 |  | FGF-R1 | 4 | 1 | 82 | 5 |
| SIK3 | 4 | 1 | 83 | 4 |  | HER4 | 4 | 0 | 84 | 9 |
| TSSK1 | 1 | 0 | 68 | 6 |  | IGF-1R | 4 | 0 | 92 | 2 |
| CK1 gamma 2 | 10 | 3 | 80 | 12 |  | IR | 2 | 0 | 100 | 3 |
| CK1 delta | 6 | 5 | 36 | 5 |  | IRR | 6 | 2 | 79 | 2 |
| CK2 | 1 | 0 | 23 | 4 |  | TrkA | 1 | 2 | 53 | 8 |
| TTBK1 | 82 | 6 | 103 | 5 |  | DDR2 | 0 | 0 | 44 | 2 |
| TTBK2 | 91 | 8 | 86 | 3 |  | VEGFR1 | 1 | 0 | 43 | 4 |
| DYRK1A | 1 | 0 | 23 | 2 |  | PDGFRA | 20 | 23 | 35 | 4 |
| DYRK2 | 1 | 0 | 18 | 1 |  | PINK | 89 | 13 | 93 | 2 |
| DYRK3 | 1 | 1 | 5 | 1 |  |  |  |  |  |  |

^a^ %AR means % kinase activity remaining.

^b^ SD means standard deviation.
